# An unusual intraoperative finding: Left atrial dissecting intramural hematoma after aortic root replacement

**DOI:** 10.1016/j.xjtc.2022.03.016

**Published:** 2022-04-18

**Authors:** Rosa Bernabeu Santisteban, Maria Victoria Johannessen López, Paula Carmona García, Iratxe Zarragoikoetxea Jauregui, Pilar Argente Navarro

**Affiliations:** Department of Anesthesiology and Critical Care, Hospital Universitari I Politècnic la Fe, Valencia, Spain

**Figure:**
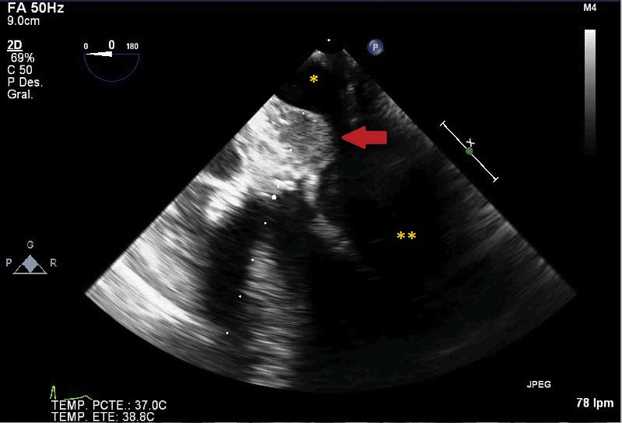
Midsesophageal 4-chamber view. LA wall intramural hematoma compressing the LA cavity.

**Central Message**

Left atrial intramural dissecting hematoma is a rare complication after cardiac surgery for which conservative treatment is feasible.

Atrial dissecting intramural hematoma is an uncommon and poorly understood complication with a controversial diagnosis and treatment. It can be found after mitral or aortic replacement, although it has also been described after percutaneous cardiac procedures, cardiopulmonary resuscitation, or spontaneously. We present the case of a young patient with Marfanoid habitus who underwent elective aortic valve and aortic root replacement secondary to ascending aortic aneurysm with the incidental finding of a dissecting intramural hematoma in the left atrial (LA) wall after cardiopulmonary bypass (CPB) disconnection.

## Case Presentation

We present the case of a 19-year-old man with Marfanoid habitus. Based on computed tomography angiogram findings of a 9.1-cm ascending aortic aneurysm, elective ascending aneurysm surgery was planned.

**Figure 1:**
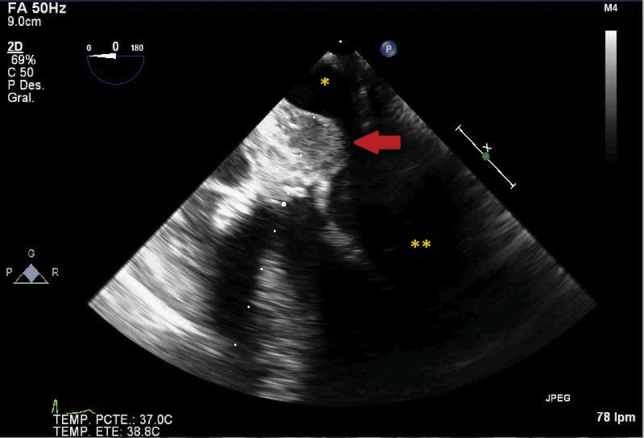
Midsesophageal 4-chamber view focused on hyperechogenic mass compatible with clotted LA wall intramural hematoma compressing the LA cavity. The *arrow* points at the image of an intramural hematoma. ∗Left atrium. ∗∗Left ventricle.

**Figure 2:**
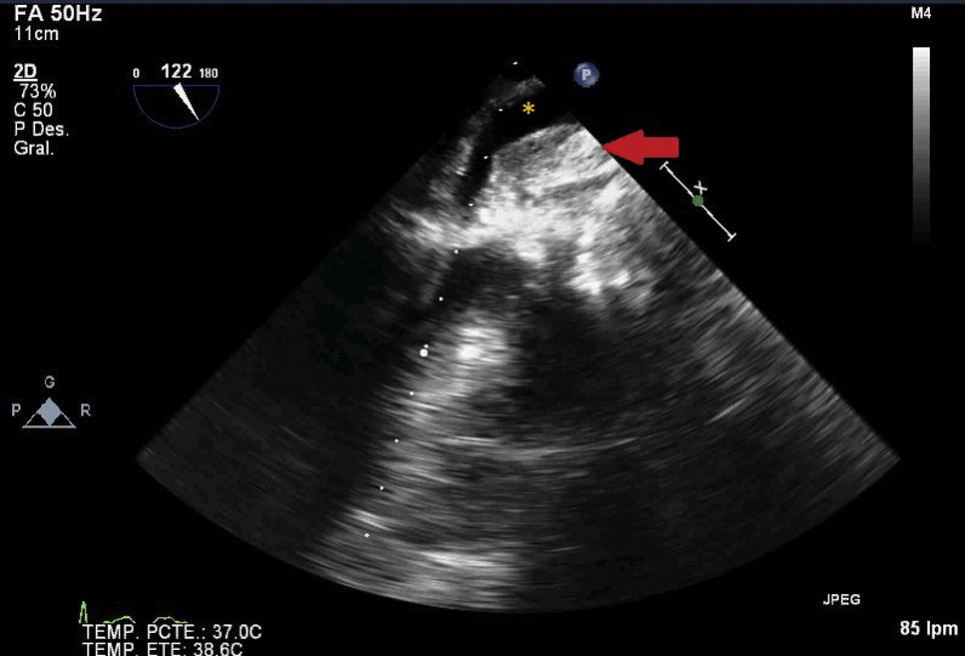
Midesophageal aortic valve long axis view. Echo dense intramural hematoma (*red arrow*) located posteriorly to the mechanical aortic valve extending towards the roof wall of the LA and compressing the LA cavity. ∗Left atrium.

**Figure E1:**
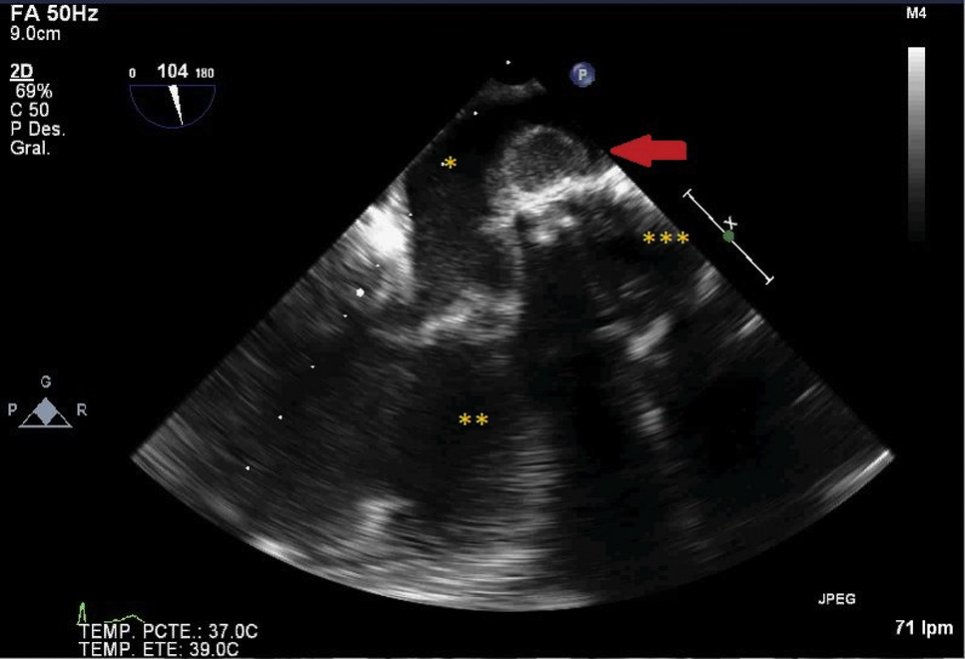
Midesophageal 3-chamber view. Echo dense intramural hematoma (*red arrow*) posteriorly located to the mechanical aortic valve protruding in the LA cavity. ∗Left atrium. ∗∗Left ventricle. ∗∗∗Aorta and aortic prosthetic valve.

**Figure E2:**
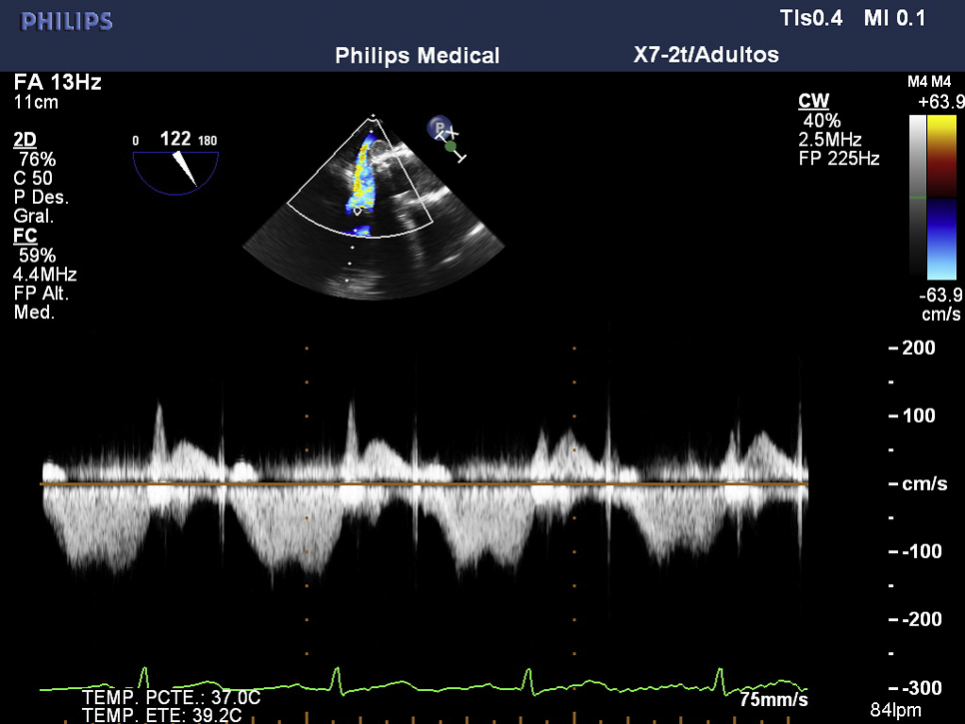
Midesophageal 3-chamber view. Continuous spectral Doppler in mitral valve showing acceleration of transmitral flow.

**Figure E3:**
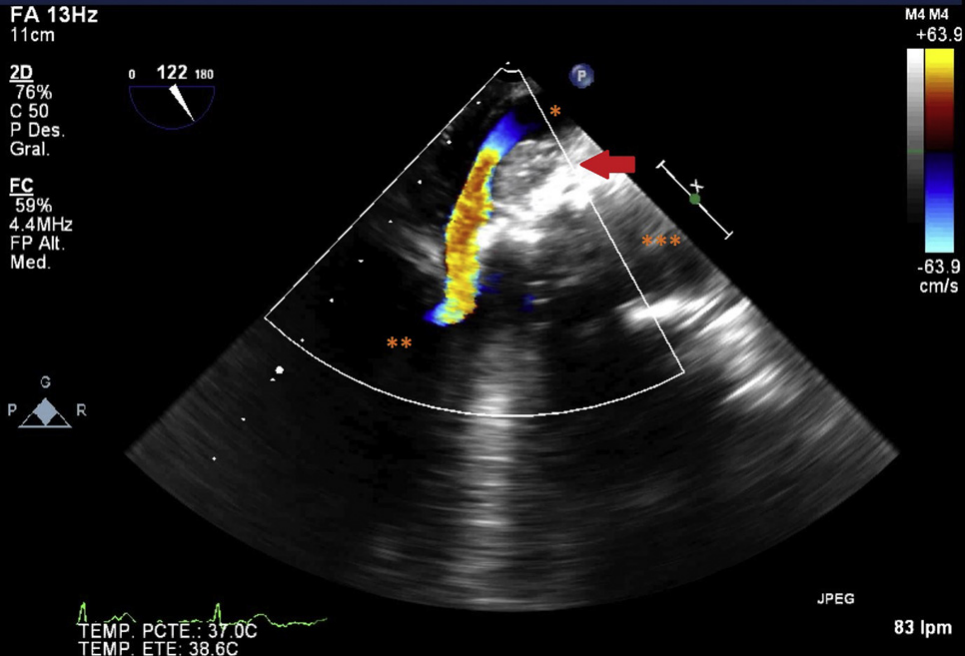
Midesophageal 3-chamber view. Echo dense intramural hematoma (*red arrow*) located posteriorly to the mechanical aortic valve protruding in the LA cavity. *Color Doppler* shows an accelerated flow in midcavity. ∗Left atrium. ∗∗Left ventricle. ∗∗∗Aorta and aortic prosthetic valve.

**Figure E4:**
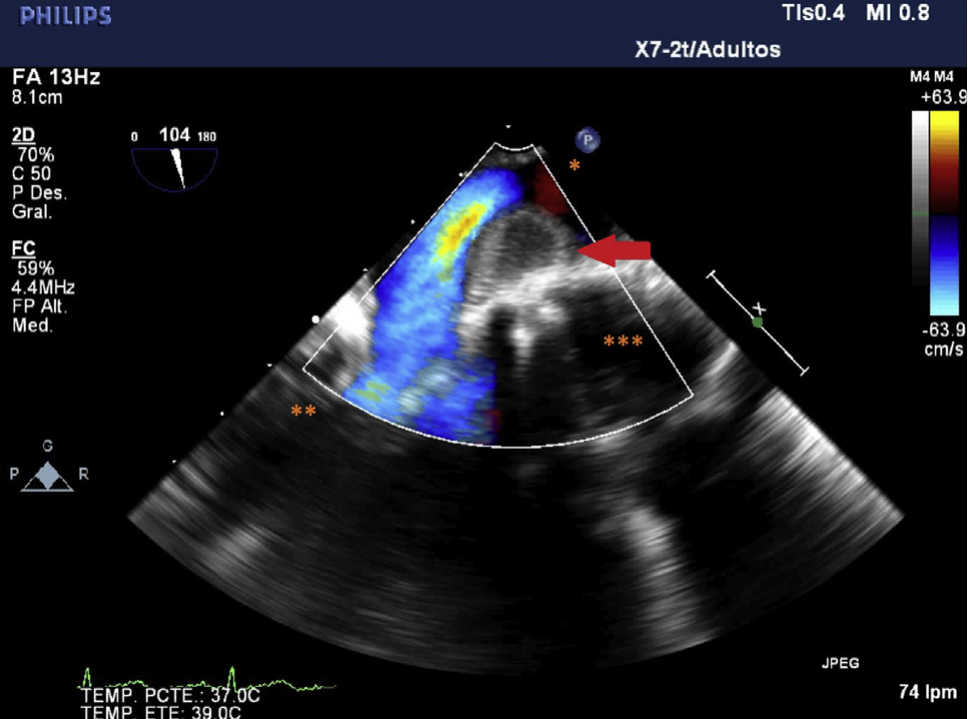
Midesophageal aortic valve long-axis view. Echo dense image (*red arrow*) posteriorly located to the mechanical aortic valve extending toward the roof wall of the LA. *Color Doppler* shows an accelerated flow in the midcavity of LA. There is no flow inside the intramural hematoma or communication with LA or pericardial space. ∗Left atrium. ∗∗Left ventricle. ∗∗∗Aorta and aortic prosthetic valve.

The intraoperative surgical findings confirmed the presence of a chronic ascending aorta dissection. Hemiarch and ascending aorta with valve-sparing root replacement was performed. Transesophageal echocardiography (TEE) showed significant residual aortic regurgitation; therefore, a second entrance in CPB was needed for mechanical aortic valve replacement (AVR). TEE examination after the second disconnection of CPB (total time 316 minutes) showed severe biventricular systolic dysfunction, well-positioned and functioning mechanical aortic valve prosthesis, and a mild anterior periprosthetic leak. An hyperechogenic mass (2 cm × 2 cm) (Figure 1, Figure 2, E1 and E2) was found between the posterior wall of the aortic graft and the roof of the LA, conditioning compression and an accelerated transmitral inflow (Figure E3). Neither flow inside the mass nor communication with surrounding structures was observed (Figure E4).

Echocardiographic findings suggested the presence of a dissecting and intramural hematoma in the LA wall related to the surgery. Giving the absence of severe hemodynamic impact of the hematoma, contained intramural location, absence of flow or communication with other structures, and no external identification after manual inspection, the surgical team maintained a conservative approach.

During the first postoperative days, an improvement of the systolic dysfunction was observed. Day 7 TEE showed a smaller dissecting intramural hematoma, moderate left pleural collection, and hemopericardium without signs of hemodynamic compromise. On day 16, TEE showed an organized pericardial collection without presence of the intramural hematoma. The patient was discharged from hospital 19 days after the surgery with a left ventricular ejection fraction of 59%. Two months later, he was reoperated due to an anterior pseudoaneurysm detected in an echocardiographic examination. A bleeding point in right coronary artery suture was identified as the origin of the pseudoaneurysm with no relation or connection with the posterior intramural hematoma identified in the first surgery.

The institutional review board or equivalent ethics committee of the Hospital Universitari i Politècnic la Fe expressed it was unnecessary to approve this case report by the ethics committee to be published. Patient written and oral consent for the publication of the study was expressed.

## Discussion

The presence of a hyperechogenic mass in the LA in this type of surgery may allow differential diagnosis between hematoma in the posterior wall of the aortic graft, pericardial collection, hematoma in the atrioventricular groove, and LA wall dissection. All of them are usually detected after discontinuation of CPB.^1^^,^^2^

They are rare complications that can be found after valve surgery, percutaneous cardiac procedures, cardiopulmonary resuscitation, or spontaneously.^1^, ^2^, ^3^, ^4^ Anatomically, the division of the noncoronary aortic annulus allows entry into the LA wall. A failed attempt of aortic valve–sparing surgery and AVR may cause aortic annular disruption, which might result in atrial wall dissection and contained hematoma.^5^ Another potential mechanism explaining this finding is the difficulty placing the retrograde cannula in the coronary sinus, which in our case was atraumatic according to surgeons' perspective.

Left atrial wall dissecting hematoma diagnosis and management may be challenging. TEE is the technique of choice in the intraoperative and postoperative scenario, and its treatment usually depends on the patient's hemodynamic stability and hematoma's progression. The presence of blood flow into the hematoma, active bleeding, size progression, severe LA compression, or prosthesis dysfunction might be criteria for surgical approach.^2^^,^^5^^,^^6^ Many cases of benign course of LA intramural hematoma with conservative treatment have been reported.^3^^,^^7^

A hematoma in LA wall could lead to functional mitral stenosis, impairment of pulmonary venous return, compression of pulmonary artery, and thereby failure to wean off CBP.^1^^,^^2^^,^^5^^,^^6^ Our patient had a contained dissecting intramural hematoma in the LA wall roof with a moderate compression of LA, which presumably in later days drained causing a hemopericardium and pleural effusion. This evolution could explain the findings in the echocardiographic examens performed on 7th and 16th postoperative days in which a decrease of dissecting hematoma and a significant pericardial and pleural effusion were observed.

## Conclusions

In summary, herein we present rare images and an uncertain and infrequent evolution of an intraoperative dissecting LA intramural hematoma after AVR and aortic root and hemiarch replacement. Although controversial, the LA intramural dissecting hematoma conservative approach is feasible, but it requires close patient surveillance.
